# MicroscopyGPT: Generating Atomic-Structure Captions from Microscopy Images of 2D Materials with Vision-Language Transformers

**DOI:** 10.1021/acs.jpclett.5c01257

**Published:** 2025-07-01

**Authors:** Kamal Choudhary

**Affiliations:** † Material Measurement Laboratory, 10833 National Institute of Standards and Technology, Gaithersburg, Maryland 20899, United States; ‡ Department of Electrical and Computer Engineering, Whiting School of Engineering, Johns Hopkins University, Baltimore, Maryland 21218, United States; § Department of Materials Science and Engineering, Whiting School of Engineering, Johns Hopkins University, Baltimore, Maryland 21218, United States

## Abstract

Determining complete atomic structures directly from microscopy images remains a long-standing challenge in materials science. MicroscopyGPT is a vision-language model (VLM) that leverages multimodal generative pretrained transformers to predict full atomic configurations, including lattice parameters, element types, and atomic coordinates, from scanning transmission electron microscopy (STEM) images. The model is trained on a chemically and structurally diverse data set of simulated STEM images generated using the AtomVision tool and the JARVIS-DFT as well as the C2DB two-dimensional (2D) materials databases. The training set for fine-tuning comprises approximately 5000 2D materials, enabling the model to learn complex mappings from image features to crystallographic representations. I fine-tune the 11-billion-parameter LLaMA model, allowing efficient training on resource-constrained hardware. The rise of VLMs and the growth of materials data sets offer a major opportunity for microscopy-based analysis. This work highlights the potential of automated structure reconstruction from microscopy, with broad implications for materials discovery, nanotechnology, and catalysis.

## Graphical abstract

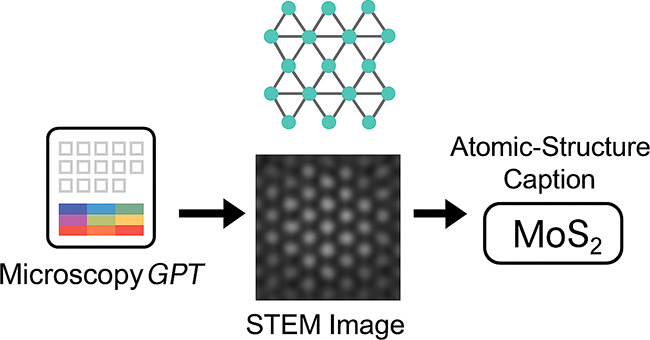

Since the development of the electron microscope in the 1930s, electron-based imaging techniques have revolutionized our ability to visualize matter at the nanoscale and beyond.^1^ Due to their significantly shorter wavelengths, up to 100 000 times smaller than those of visible light, electrons enable imaging at remarkably higher spatial resolutions. Key microscopy techniques, including scanning transmission electron microscopy (STEM), transmission electron microscopy (TEM), atomic force microscopy (AFM), and scanning tunneling microscopy (STM), have become indispensable tools for studying material properties at the atomic scale.^1,2^

Among the diverse branches of electron microscopy, STEM has emerged as a critical tool in materials science, nanotechnology, and structural biology due to its unparalleled spatial resolution and multimodal imaging capabilities.^1,3^ STEM is widely applied to investigate lattice defects, interfaces, phase transformations, and chemical heterogeneities with atomic precision.^4,5^ Unlike conventional imaging methods, STEM enables point-by-point scanning of a focused electron probe across a thin specimen, collecting transmitted signals, such as bright-field (BF), annular dark-field (ADF), and high-angle annular dark-field (HAADF) images. These channels provide complementary contrast mechanisms based on the atomic number, thickness, and crystallographic orientation.^6^ Furthermore, integration with electron energy loss spectroscopy (EELS) and energy-dispersive X-ray spectroscopy (EDS) extends STEM into a powerful analytical platform for quantitative chemical and electronic characterization at the atomic scale.^7^

Despite these remarkable advancements, interpreting STEM images to extract complete 3D atomic structures remains a challenging and largely manual process. The inverse problem of reconstructing atomic coordinates from projected 2D contrast patterns is inherently ill-posed, requiring significant domain expertise, sophisticated image preprocessing, and iterative fitting procedures using physical simulations. This complexity presents a substantial bottleneck in accelerating materials discovery and implementing high-throughput characterization workflows. The challenge is further amplified by experimental variations in imaging conditions, sample orientation, thickness fluctuations, and instrumental noise, which collectively complicate direct interpretation and structure retrieval from STEM images.

Recent developments in machine learning have shown considerable promise in automating and accelerating materials characterization tasks.^8–10^ Several pioneering efforts have addressed inverse design and inverse learning problems using microscopy images. For instance, De Backer et al. developed a Bayesian genetic algorithm framework for reconstructing atomic models of monotype crystalline nanoparticles from single *Z*-contrast projections.^11^ Deng et al. introduced a physically constrained image-learning approach to algorithmically derive chemo-mechanical constitutive laws at the nanoscale.^12^ Lin et al. created TEMImageNet and AtomSegNet to perform robust and precise atom segmentation, localization, denoising, and super-resolution processing of experimental images.^13^ Comprehensive reviews by Kalinin et al. document numerous advances in applying machine learning techniques to STEM image analysis and interpretation.^14,15^ More detailed reviews on the application of deep learning in microscopy can be found elsewhere.^16,17^

Materials data can be broadly classified into four categories: (a) scalar data, such as electronic bandgap values, (b) spectral or multivalue data, including diffraction patterns, (c) image data from various microscopy techniques, and (d) textual data from scientific literature.^8^ Recent models, such as Atomistic Generative Pretrained Transformer (AtomGPT)^18^ and DiffractGPT,^19^ have demonstrated the transformative potential of generative AI for processing scalar and multivalue data sets, respectively, establishing crucial bridges between atomic structures and experimental measurements. AtomGPT specializes in predicting material properties or generating atomic configurations from specified scalar inputs, while DiffractGPT extends this framework to diffraction patterns, enabling crystal structure determination from X-ray diffraction (XRD) data. These innovations highlight the versatility of transformer-based architectures in handling diverse types of material data and addressing complex structure–property relationships. Notably, although XRD provides valuable averaged structural information, it cannot directly visualize individual defects, interfaces, dislocations, dopants, or structural variations across interfaces, capabilities that are uniquely enabled by transmission electron microscopy techniques.

Building upon these foundations, I introduce MicroscopyGPT, a novel framework that extends the transformer-based generative approach to microscopy image data sets. The system employs the powerful 11-billion-parameter Large Language Model Meta AI (Llama)^20^ vision-language model architecture, fine-tuned using Quantized Low-Rank Adaptation (QLoRA), to efficiently map microscopy images directly to their corresponding atomic structures. This approach eliminates the need for iterative manual fitting procedures, enabling the automated and accurate determination of atomic arrangements from complex and often noisy microscopy data. MicroscopyGPT is specifically designed to address the unique challenges associated with image-based structural determination, complementing and extending the capabilities of AtomGPT and DiffractGPT to a new experimental domain.

The MicroscopyGPT framework offers several key advantages over conventional approaches: (1) direct end-to-end mapping from microscopy images to complete atomic structures, (2) ability to integrate multimodal information (textual and visual) for improved accuracy, (3) extensibility to diverse material systems beyond the training distribution, and (4) compatibility with physics-informed refinement techniques for enhanced structural fidelity.

As experimental and computational data sets continue to evolve, the framework can be readily extended to encompass new materials systems, imaging modalities, and generative model architectures, paving the way for broader applications in catalysis, semiconductor technology, energy materials, and nanotechnology. To promote reproducibility and facilitate further development, the complete codebase used in this study will be made available on the AtomGPT GitHub repository: https://github.com/atomgptlab/atomgpt.

The foundation of this work is the 2D materials data sets JARVIS-DFT-2D and the Computational 2D Materials Database (C2DB), which are comprehensive repositories containing approximately 1103 and 3520 atomic structures, respectively, along with associated material properties calculated using density functional theory (DFT).^21,22^ There are various databases that contain STEM and atomic structure information.^8^ However, in this work, I choose to use the above publicly available data sets for proof of concept applications. Note that, although a simulated STEM database is used here, it can be easily extended to include experimental data in the future.

To develop a machine-learning-compatible data set, I employed the AtomVision^23^ framework to generate high-resolution STEM images that accurately simulate experimental conditions. The STEM image simulation process^24–28^ utilized a convolution-based approach founded on fast Fourier transform principles, mathematically expressed as

1 I(r)=R(r,Z)⊗PSF(r)

where *r* represents a two-dimensional vector in the image plane, *I*(*r*) denotes the image intensity, and PSF­(*r*) is the microscope’s point spread function. The transmission function *R*(*r*, *Z*) incorporating the atomic potential information is defined as

2 $$R\left(r,Z\right)=\overset{N}{\underset{i}{\sum }}Z_{i}^{1.7}\delta \left(r-r_{i}\right)$$

This function aggregates contributions from *N* atoms positioned at coordinates *r*_*i*_, with *Z*_*i*_ representing the atomic number of each atom. While the Rutherford scattering theory predicts a *Z* ^2^ dependence of scattered intensity, the effective exponent is reduced to 1.7 due to core electron screening effects and the influence of detection collection angles. Previous research has employed power values ranging from 1.3 to 1.7 to achieve optimal alignment with experimental observations.^29^ For consistency across diverse material systems, I standardized an exponent value of 1.7 throughout the simulations.

Each simulated STEM image was generated at a resolution of 256 × 256 pixels, capturing a physical area of at least 2.5 × 2.5 nm. The microscope’s point spread function was modeled as a normalized Gaussian with a characteristic width of 0.5 Å, approximating the resolution limitations of state-of-the-art aberration-corrected STEM instruments. For every 2D material in the data set, I generated STEM images along the common Miller index (001), providing a consistent crystallographic projection for model training.

To enhance the realism of the simulated data set, I systematically introduced controlled variations that replicate common experimental artifacts: (1) Gaussian noise simulates electronic detector noise, thermal fluctuations, and quantum shot noise inherent in experimental microscopy; (2) Gaussian blur accounts for residual lens aberrations, finite probe size effects, and specimen drift during image acquisition; and (3) intensity variations were incorporated to mimic beam damage, scanning distortions, and local thickness variations. Note that Gaussian noise alone may still not be enough to precisely mimic the noise status in the experimental STEM imaging.^30^

These augmentations collectively attempt to ensure that the simulated images closely resemble the imperfections encountered under real experimental STEM imaging conditions. The augmented STEM images were paired with their corresponding structural metadata, including lattice parameters, lattice angles, element types, and fractional atomic coordinates, to form comprehensive training examples for the machine learning model.

The MicroscopyGPT framework leverages the LLaMA-3.2-11B-Vision-Instruct architecture, a state-of-the-art multimodal large language model (MLLM) designed to process atomic resolution microscopy images in conjunction with structured textual prompts. The model is based on an 11-billion-parameter decoder-only transformer derived from Meta’s LLaMA-3.2 family and enhanced with a high-capacity vision encoder, enabling it to extract crystallographic information from high-resolution STEM images. MicroscopyGPT operates on multimodal inputs composed of an image and a natural language prompt. The model uses the processor and tokenizer from the Llama-3.2–11b-vision-instruct-unsloth-bnb-4bit checkpoint. The textual input is tokenized using a fast SentencePiece-based tokenizer with a vocabulary of 128 257 tokens. This vocabulary includes standard subword units and special tokens, such as <|begin_of_text|>, <|eot_id|>, and <|image|>, along with over 240 reserved tokens used for internal instruction formatting. The tokenizer supports sequences up to 131 072 tokens and applies right padding for alignment during training and inference. The image input is processed using a Vision Transformer (ViT-H) that acts as the visual tokenizer. Each microscopy image is resized to 560 × 560 pixels, converted to RGB (if needed), normalized with ImageNet-standard channel statistics, and rescaled by a factor of 1/255. The normalized image is partitioned into 256 non-overlapping patches, which are converted into 1024-dimensional visual tokens using the ViT-H encoder. These patch tokens are inserted between the special tokens <|image_start|> and <|image_end|> to mark the beginning and end of the visual segment in the token sequence. A typical input includes an image and a prompt such as: The chemical formula is Ni_2_Si. Generate an atomic structure description with lattice lengths, angles, coordinates, and atom types. Also predict the Miller index. The model produces outputs in the form of plain text predictions that encode structural information, e.g., 3.92 3.92 5.02 90 90 120 Si 0.667 0.333 0.750 ... The Miller index is (0 0 1). The output contains the predicted lattice constants (in Å), lattice angles (in degrees), atom types, fractional coordinates, and Miller index derived from the paired visual and textual context. At present, symmetry operations, such as space-group generators or Wyckoff positions, are not explicitly included in the prompts. Instead, the model infers symmetry features implicitly from both image and descriptors, such as the chemical formula and one among five Bravais lattices (0, hexagonal; 1, square/tetragonal; 2, rectangle/orthorhombic; 3, rhombus/centered orthorhombic; and 4, parallelogram/monoclinic). However, the model architecture supports the inclusion of such structured symmetry data as part of the prompt, and we plan to explore this in future work.

The core language model is a 40-layer decoder-only transformer with a hidden size of 4096, a feed-forward network expansion ratio of 16/3, and 32 attention heads. This configuration supports a context window of up to 128 000 tokens, allowing for simultaneous processing of high-resolution image tokens and lengthy scientific text prompts. The vision component consists of a 32-layer vision transformer huge (ViT-H) encoder followed by an 8-layer global transformer module that aggregates and refines the visual features.

Fusion of the visual and textual modalities occurs within the decoder layers via cross-attention. Let $V\in R^{N_{v}\times d}$ denote the visual token embeddings and $T\in R^{N_{t}\times d}$ denote the textual token embeddings for *d* embedding dimensions. Cross-attention for query (*Q*), key (*K*), and value (*V*) vectors is computed as

3 $$\mathrm{attention}\left(Q,K,V\right)=\mathrm{softmax}\left(\frac{QK^{T}}{\sqrt{d_{k}}}\right)V$$

where *Q* = *TW* ^*Q*^, *K* = *VW* ^*K*^, *V* = *VW* ^*V*^, and *W* ^*Q*^, *W* ^*K*^, and *W* ^*V*^ are learned projection matrices.

The model is trained with an autoregressive next-token prediction objective. The loss function is defined as

4 $$L_{\mathrm{LM}}=-\overset{T}{\underset{i=1}{\sum }}\log P_{\theta }\left(w_{i}|w_{<i},V\right)$$

where *w*_*i*_ is the *i*th token in the output sequence, *V* represents the image-derived context, and the symbol θ denotes the set of all trainable parameters in the model. These include the weights and biases of the transformer layers, such as the self-attention projections (*W* ^*Q*^, *W* ^*K*^, and *W* ^*V*^), feedforward network weights, embedding matrices for both text and image tokens, and layer normalization parameters. The objective is to maximize the likelihood *P*_θ_(*w*_*i*_|*w*_<*i*_, *V*) of the next token *w*_*i*_ given the preceding tokens *w*_<*i*_ and the visual context *V*, which is encoded from the input microscopy image. During training, the model parameters θ are updated via back-propagation to minimize the negative log-likelihood across the target sequence, enabling the model to learn a joint distribution over image and text modalities. This setup enables the model to learn a joint distribution over text and microscopy imagery, facilitating accurate atomic structure generation from experimental and synthetic inputs.

To adapt the pretrained Llama model to the specialized task of atomic structure inference from STEM images, I employed the QLoRA method.^31^ This parameter-efficient fine-tuning approach significantly reduces computational requirements by introducing low-rank adapters into specific transformer layers while keeping the majority of the original model parameters fixed. Specifically, I maintained 99.7% of the parameters unchanged and only modified a small subset of parameters critical for domain adaptation.

The QLoRA implementation was facilitated through the UnslothAI package,^32^ which provides optimized routines for efficient training of large language models. The supervised fine-tuning protocol was designed to establish mapping between STEM imagery and structured textual descriptions of atomic configurations. The input STEM images were preprocessed, normalized, and tokenized as 256 × 256 grayscale inputs, while the corresponding outputs consisted of structured textual descriptions encompassing lattice parameters, lattice angles, element types and positions, fractional atomic coordinates, and the Miller index. I use a Miller index of (001) for this data set, and it can be extended for other Miller indices in the future as well.

To maintain consistent data representation across examples, all outputs were formatted according to a standardized chat template that structured the crystallographic information in a human-readable yet machine-parsable format. The training process involved minimizing the discrepancy between model-generated structural descriptions and ground-truth crystallographic data derived from the DFT databases.

The fine-tuned model was rigorously evaluated on a held-out test set comprising 10% of the original data set, which was carefully selected to ensure representation across diverse structural motifs, chemical compositions, and crystallographic complexities.

In the context of materials science, the model performs structured captioning by generating comprehensive crystallographic information from the microscopy images. This task represents an inverse problem that can be formally expressed as

5 f:I→S

where *I* denotes the input STEM image and *S* represents the structured symbolic output, including lattice parameters, fractional coordinates, and atomic elements. After structure generation, a unified graph neural network force field, such as Atomistic Line Graph Neural Network Force Field (ALIGNN-FF),^33^ can be employed as a fast post-processing tool to optimize the predicted atomic configuration.

The performance of the MicroscopyGPT model was assessed using multiple complementary metrics: (1) Earth mover’s distance (EMD) to measure the minimum “work” required to transform the predicted distribution of structural features into the ground-truth distribution, offering a robust assessment of distributional similarity, (2) Kullback–Leibler divergence (KLD) to evaluate the information–theoretic difference between predicted and ground-truth probability distributions of structural properties, capturing subtle divergences in the model’s predictions, and (3) root mean square error (RMSE) to assess the positional accuracy of atomic coordinates, calculated as the square root of the average squared displacement between predicted and reference atomic positions after optimal alignment. These diverse evaluation metrics collectively provide a comprehensive assessment of the model’s ability to reconstruct accurate atomic structures from microscopy data across various materials systems with different chemical compositions and structural complexities.

Note that this work focuses on 2D materials, primarily using STEM images along the (001) Miller index, which is typically the most relevant orientation for layered structures. The training data set includes entries from both the JARVIS-DFT-2D^34^ and C2DB^35^ databases to enhance structural diversity. While capturing symmetry-related features from multiple Miller indices is important for 3D materials, such a data set expansion is currently limited by the significant computational cost of simulating and training on large multi-orientation image sets. Future work will aim to address these limitations through high-throughput STEM simulation pipelines and more efficient vision–language transformer architectures.

The MicroscopyGPT framework introduced here demonstrates the capability for direct inference of complete atomic structures from computational STEM images. As illustrated in Figure 1, the architecture seamlessly integrates vision and language processing through a sophisticated multimodal transformer pipeline. Although it currently uses Llama-3.2-11B-Vision-Instruct on the JARVIS-DFT and C2DB 2D materials data sets, it can be easily extended for other futuristic models and data sets. The system processes two primary inputs: a high-resolution STEM image and a natural language prompt specifying the chemical composition of the material (e.g., Ni_2_Si) and a prompt to generate the atomic structure. The STEM image undergoes encoding through a vision transformer, while concurrently, the textual prompt is converted into token embeddings via a specialized tokenizer. These dual representations are then fused and processed by the MicroscopyGPT transformer model, which generates a comprehensive crystallographic representation encompassing lattice parameters, atomic positions, elemental types and coordinates, and a predicted Miller index.

**Figure 1:**
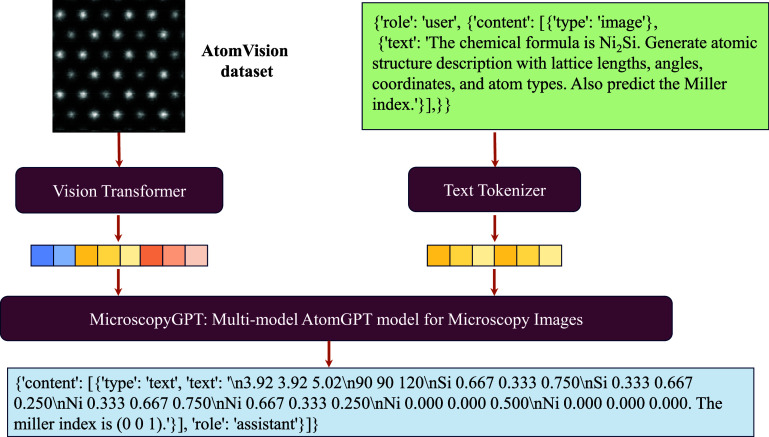
Overview of the MicroscopyGPT framework for atomic structure inference from STEM images. The model receives a high-resolution STEM image alongside a natural language prompt describing the material system (e.g., Ni_2_Si). A vision transformer encodes the microscopy image, and a tokenizer converts the textual prompt into token embeddings. These are jointly processed by a multimodal transformer (MicroscopyGPT) to generate a complete atomic structure description, including lattice parameters, angles, atomic positions, element types, and predicted Miller index. Such training data are developed using the AtomVision tool for simulated STEM images of 2D materials from the JARVIS-DFT database.

This end-to-end approach eliminates the need for traditional manual fitting procedures or computationally expensive physical simulations. The foundation of the model’s training lies in the synthetically generated STEM images derived from the AtomVision pipeline, which leverages the structurally and chemically diverse data set. This strategic pairing of simulation-based data augmentation with advanced generative modeling enables direct atomic-level prediction, representing a significant advancement in automated crystallographic analysis from microscopy data.

To assess the performance of the model, a comprehensive quantitative evaluation was carried out on the held-out 10% test set comprising diverse crystalline materials of both the JARVIS-DFT and C2DB data sets. Figure 2 presents a detailed comparison between predicted and target structural and chemical property distributions for the C2DB data set. Panels a–c show histograms of lattice parameters *a* and *b* (measured in Å) and the γ angle (in degrees), which resemble each other. Similarly, panels d and f showcase the distributions of space group numbers, Bravais lattice types, and total atomic weights (in atomic mass unit, 1 AMU = 1.66 × 10^–27^ kg), respectively. The training data set shows a strong peak near 4 Å for both *a* and *b* lattice parameters, as seen in Figures S1 and S2 of the Supporting Information. This also reflects in panels a–c. There are 5 Bravais lattices in 2D: 0, hexagonal; 1, square/tetragonal; 2, rectangle/orthorhombic; 3, rhombus/centered orthorhombic; and 4, parallelogram/monoclinic. The close correspondence between predicted and target distributions across these parameters underscores MicroscopyGPT’s ability to infer fundamental crystallographic and compositional characteristics from STEM images. The histograms are normalized to reflect the material frequency across property bins, providing a statistically sound basis for comparison. A similar analysis for the JARVIS-DFT-2D data set is available in Figure S3 of the Supporting Information.

**Figure 2:**
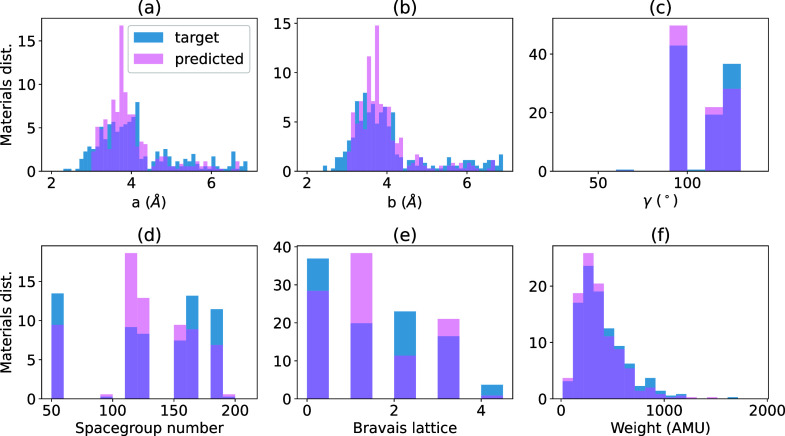
Comparison of predicted and target structural properties on the test data set using the MicroscopyGPT model for C2DB data set. Panels a–c show histograms of lattice parameters *a* and *b* (in Å) and γ (in degree), while panels d–f depict distributions of space group numbers, Bravais lattice types, and total atomic weight (in AMU), respectively. Predicted values closely follow the target distributions, indicating the model’s ability to accurately infer key crystallographic and compositional features from STEM images. Histograms are normalized to reflect material frequency across property bins.

For a more granular assessment of the distributional alignment between predicted and ground-truth structural properties, Table 1 provides quantitative metrics, including the range (minimum and maximum values) for each parameter alongside two statistical measures of distribution similarity: Kullback–Leibler divergence (KLD) and Earth mover’s distance (EMD). Lower values of both KLD and EMD indicate higher similarity between distributions. Notably, the model demonstrates particularly strong performance in predicting lattice angles (γ) with a minimal KLD of 0.02 and EMD of 0.54 while showing slightly higher divergence in space group prediction (KLD = 0.72 and EMD = 3.24) for JARVIS-DFT-2D. Model trained on JARVIS-DFT shows higher divergence in space group and lattice parameters, while the model trained on C2DB performs better on those but worse on γ. These metrics provide a rigorous statistical foundation for evaluating the model’s predictive accuracy across different structural parameters.

**Table 1:** Predicted and Target Values for Structural Properties in the JARVIS-DFT 2D and C2DB Data Sets^a^

	JARVIS-DFT 2D data set				C2DB data set			
property	min	max	KLD	EMD	min	max	KLD	EMD
*a* (Å)	2.38	14.40	0.06	1.67	2.39	10.47	0.02	0.69
*b* (Å)	2.51	15.64	0.07	0.91	2.40	10.46	0.02	0.72
γ (deg)	60.0	120.0	0.02	0.54	60.0	120.0	0.01	1.94
space group	1	191	0.72	3.24	1	191	0.52	7.00
weight (AMU)	24.82	2958.62	0.11	1.08	26.02	1661.16	0.03	1.69

a Lower KLD and EMD values indicate better alignment. The model trained on JARVIS-DFT shows higher divergence in space group and lattice parameters, while the model trained on C2DB performs better on those but worse on angle γ.

For an in-depth qualitative assessment, Figure 3 presents detailed case studies of three representative materials from the test set: ScBr (space group *P*4/*nmm*), YCl_2_ (space group *P*6̅*m*2), and Pt_5_AlCl (space group *Amm*2) with varying RMSE values. Lower RMSE values represent better predictions. Each row displays a comprehensive comparison between the input STEM image, the target atomic structure, and the MicroscopyGPT-generated structure, providing a quantitative analysis of the model’s predictive capabilities across materials with varying chemical compositions and crystallographic complexities.

**Figure 3:**
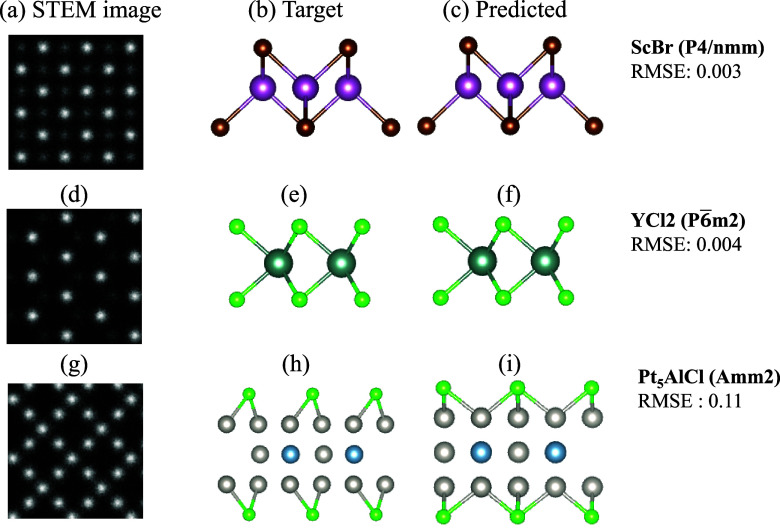
Evaluation of MicroscopyGPT for atomic structure predictions. Examples from the test set are shown for three materials: (a, b, and c) ScBr (*P*4/*nmm*, (d, e, and f) YCl_2_ (*P*6̅*m*2), and (g, h, and i) Pt_5_AlCl (*Amm*2). Each row contains the STEM image and target and generated structures. RMSE values quantify deviations between the predicted and ground-truth atomic positions.

Figure 4 shows the application of MicroscopyGPT on three experimental STEM images of layered compounds: graphene, MoS_2_, and FeTe. The model is able to predict reasonable atomic structures for graphene and MoS_2_, while the FeTe case exhibits some deviation from its experimentally known structure, potentially due to the structural complexity or image quality. Although the current model is trained primarily on simulated data for 2D materials, this preliminary evaluation demonstrates promising generalizability to real-world images. Across all three case studies, the predicted structures exhibit good agreement with the ground truth in terms of both symmetry and composition. For materials with a greater number of atomic layers, the model might struggle to resolve the atomic structure because of the overleaping signals for the atoms. Applying MicroscopyGPT to bulk 3D materials remains a key direction for future work. However, the lack of chemically and structurally diverse, high-quality 3D STEM data sets with annotated structures and multiple projections presents a major challenge. Unlike the relatively abundant and well-curated data sets for 2D systems, 3D STEM image repositories are still scarce. Systematic data set curation will be essential for enabling general-purpose inverse models for microscopy-based structure prediction.

**Figure 4:**
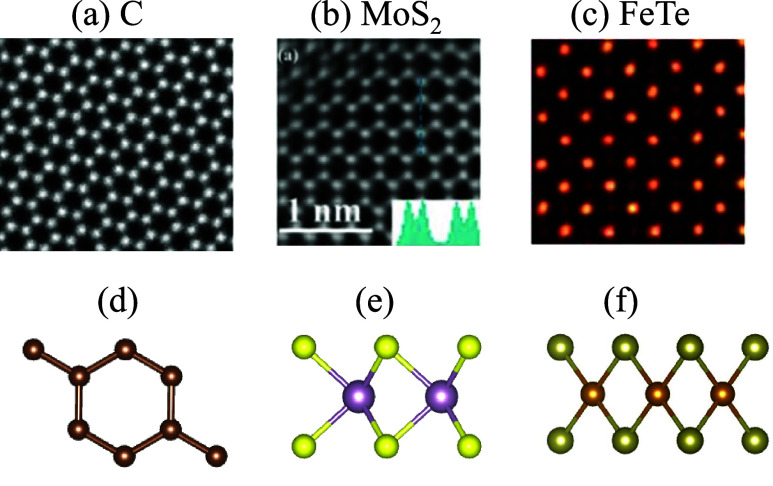
Inference on experimental STEM images for (a) graphene [This image was reproduced with permission from ref 36. Copyright 2021 O’Leary et al. Authors under Creative Commons Attribution 4.0 International License (CC BY 4.0)], (b) MoS_2_ [This image was reproduced with permission from ref 37. Copyright 2016 AIP Publishing], and (c) FeTe [This image was reproduced with permission from ref 38. Copyright 2021 Kang et al. Authors under Creative Commons Attribution 4.0 International License (CC BY 4.0)], with the corresponding generated atomic structures shown in panels d, e, and f, respectively.

The integration of vision-language models with conventional physics-based refinement offers a promising paradigm for bridging the gap between experimental observation and structural determination. This approach could substantially accelerate the materials discovery pipeline by reducing the time and expertise required for the structure solution from microscopy data.

Note that only image to structure might not be a one-to-one mapping, but adding other features in the prompt, such as Bravais lattice, chemical formula, and other experimental measurements, can augment the structure resolution process. Looking forward, several promising avenues exist for extending the capabilities of the MicroscopyGPT framework: (1) incorporation of 3D STEM tomography data to enable direct prediction of complex three-dimensional structures, (2) integration of complementary spectral information, such as EELS and EDS, to enhance chemical specificity, (3) expansion of the training data set to encompass a broader range of crystalline and non-crystalline materials, (4) development of more sophisticated vision-language models with enhanced multimodal reasoning capabilities, (5) implementation of active learning protocols to continuously refine predictions based on the experimental feedback, (6) extension to other microscopy modalities beyond STEM, including AFM and STM. (7) Currently, the data set comprises ideal 2D materials without structural imperfections. In future work, we plan to expand the training set to include defects, containing structures such as vacancies, dislocations, and grain boundaries. Notably, the AtomVision package already supports the generation of such defective structures, which can be readily integrated into future versions of the data set to improve the model’s robustness and realism. (8) Also, currently, we do not provide any symmetry information explicitly, but it can be added to the prompt. There is a potential for prompt engineering for such problems. (9) While the model demonstrates strong performance across diverse examples, a systematic ablation study on the influence of textual prompt variation and model parameters is left for future work. Such a study would help quantify the model’s robustness and interpretability, especially in cases where multiple plausible outputs may arise from the same microscopy image. Due to the high computational cost associated with running large-scale multimodal experiments, this remains an open avenue for follow-up research. Moreover, while the current study focuses on multimodal (image + text) inputs, evaluating the individual contributions of each modality remains an important area for future research. Prior studies, such as VisionLLaMA,^39^ have highlighted the performance gains achieved through multimodal integration over single-modality approaches.^40^ We plan to conduct a systematic ablation study to assess the specific impact of vision-only, text-only, and combined inputs on the model’s performance in future work.

By addressing these future directions, the MicroscopyGPT framework has the potential to evolve into a comprehensive platform for automated structural characterization across the materials science domain, significantly accelerating the pace of materials discovery and optimization.

## Supplementary Material



## Notes

Feature distribution in the JARVIS-DFT-2D data set, feature distribution in the C2DB data set, and comparison of predicted and target structural properties on the JARVIS-DFT-2D test data set (PDF)
