# Supplementary material for: MicroscopyGPT: Generating Atomic-Structure Captions from Microscopy Images of 2D Materials with Vision-Language Transformers
Source: J Phys Chem Lett. 2025 Jul 1;16(27):7028–35. doi: 10.1021/acs.jpclett.5c01257 (PMC12257582; doi:10.1021/acs.jpclett.5c01257)
Supplement: Supplementary file 1 [file jz5c01257_si_001.pdf]

Supplementary Information: MicroscopyGPT: Generating Atomic-Structure Captions from Microscopy Images of 2D Materials with Vision-Language Transformers

Kamal Choudhary[*,†,‡,¶]

†*Material Measurement Laboratory, National Institute of Standards and Technology, Gaithersburg, MD 20899, USA*

‡*Department of Electrical and Computer Engineering, Whiting School of Engineering, The Johns Hopkins University, Baltimore, MD 21218, USA*

¶*Department of Materials Science and Engineering, Whiting School of Engineering, The Johns Hopkins University, Baltimore, MD 21218, USA*

E-mail: kamal.choudhary@nist.gov

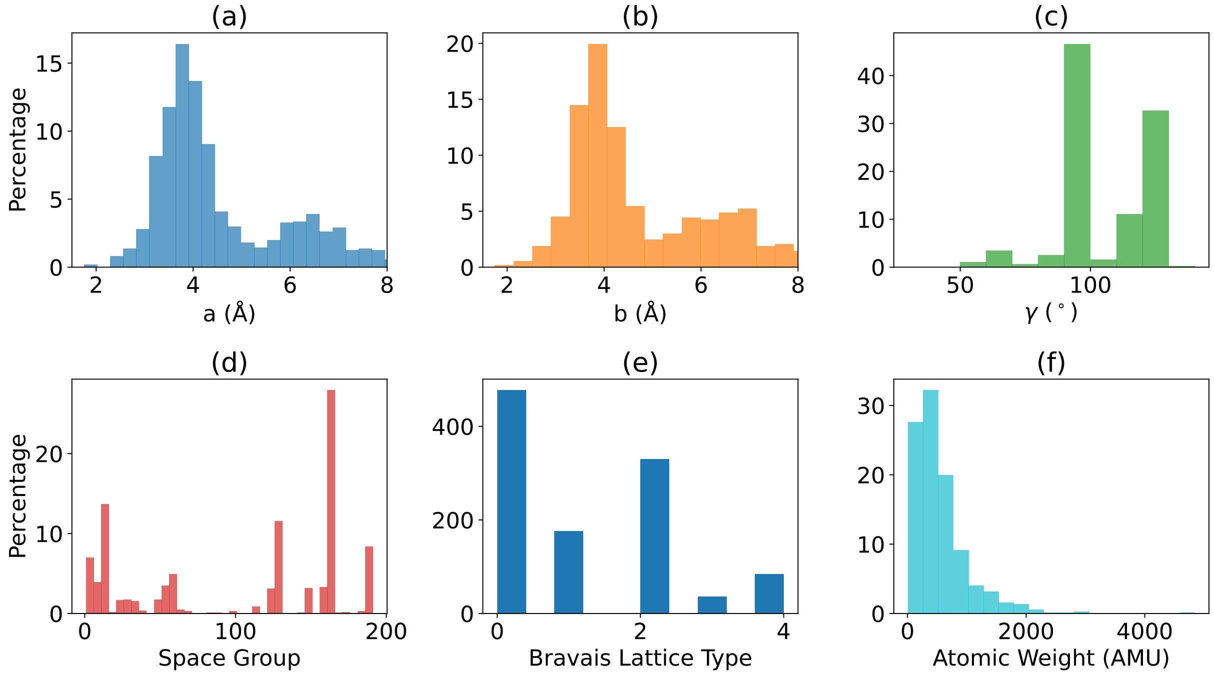

Figure S1: Feature distribution in the JARVIS-DFT-2D dataset. Subplots show histograms of (a) lattice parameter a, (b) lattice parameter b, (c) lattice angle γ, (d) space group numbers, (e) Bravais lattice types (five total in 2D), and (f) atomic weight of the constituent elements. These plots illustrate the diversity in lattice geometry, symmetry, and composition present in the dataset used to train and evaluate MicroscopyGPT.

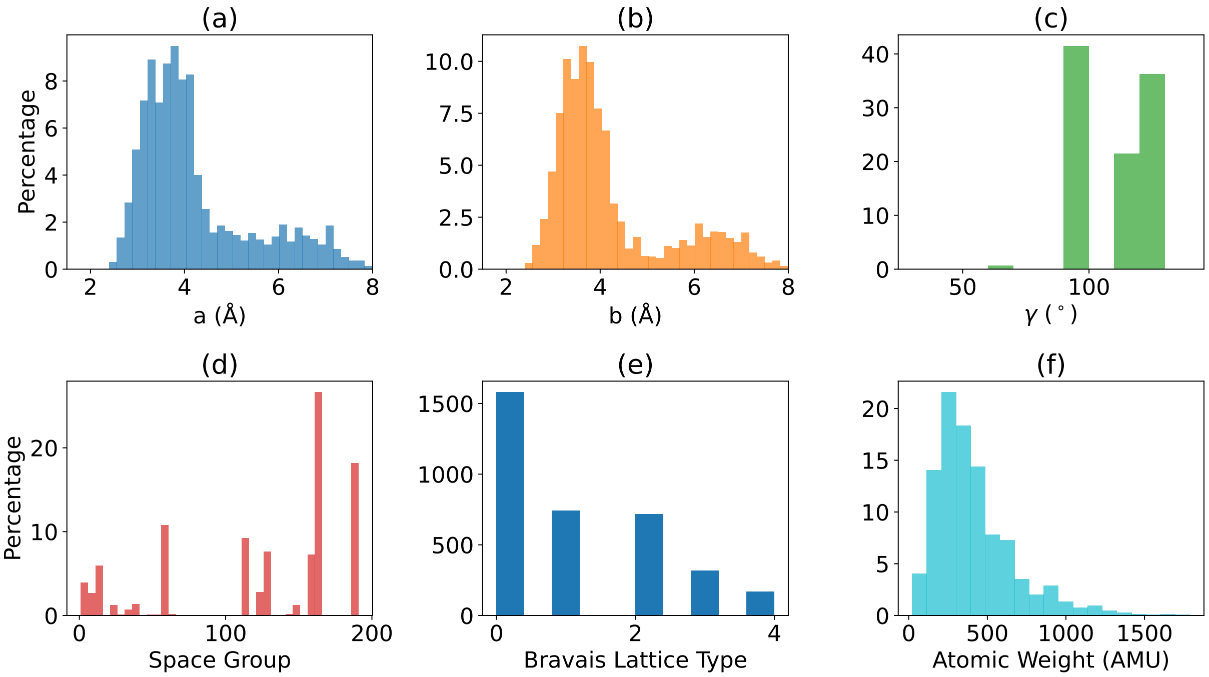

Figure S2: Feature distribution in the C2DB dataset. Subplots show histograms of (a) lattice parameter a, (b) lattice parameter b, (c) lattice angle γ, (d) space group numbers, (e) Bravais lattice types (five total in 2D), and (f) atomic weight of the constituent elements. These plots illustrate the diversity in lattice geometry, symmetry, and composition present in the dataset used to train and evaluate MicroscopyGPT.

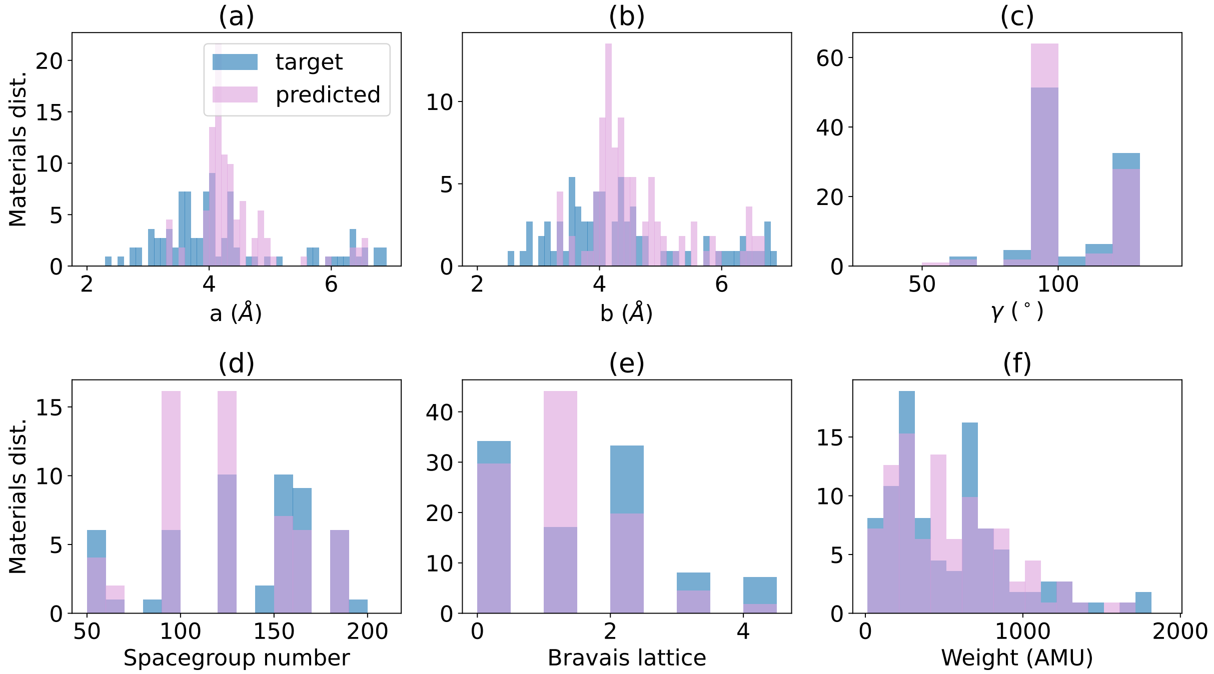

Figure S3: Comparison of predicted and target structural properties on the test dataset using the MicroscopyGPT model for JARVIS-DFT-2D dataset.(a-c) show histograms of lattice parameters a, b (in Å) and γ (in degree), while (d-f) depict distributions of space group numbers, Bravais lattice types, and total atomic weight (in AMU), respectively.
